# Lenvatinib Plus Pembrolizumab versus Doxorubicin for Advanced or Recurrent Endometrial Cancer with Short Treatment-Free Intervals Following First-Line Carboplatin Plus Paclitaxel

**DOI:** 10.3390/jcm13195670

**Published:** 2024-09-24

**Authors:** Shao-Jing Wang, Hsin-Hua Chen, Lou Sun, Yu-Hsiang Shih, Ting-Fang Lu, Yen-Fu Chen, Chun-Ting Fan, Shih-Tien Hsu, Chin-Ku Liu, Sheau-Feng Hwang, Chien-Hsing Lu

**Affiliations:** 1 Department of Gynecology and Obstetrics, Taichung Veterans General Hospital, Taichung 40705, Taiwan; intf007@vghtc.gov.tw (S.-J.W.);; 2 Division of Allergy, Immunology and Rheumatology, Department of Internal Medicine, Taichung Veterans General Hospital, Taichung 40705, Taiwan; 3 Precision Medicine Research Center, Department of Post-Baccalaureate Medicine, College of Medicine, National Chung Hsing University, Taichung 40220, Taiwan; 4 Institute of Biomedicine Science, National Chung Hsing University, Taichung 40220, Taiwan; 5 Department of Medical Research, Taichung Veterans General Hospital, Taichung 40705, Taiwan; 6 Department of Food and Nutrition, Providence University, Taichung 43330, Taiwan; 7 College of Health Care and Management, Chung Shan Medical University, Taichung 40201, Taiwan; 8 Center for General Education, Ling Tung University, Taichung 40821, Taiwan; 9 School of Medicine, China Medical University, Taichung 40604, Taiwan; 10 Department of Animal Science and Biotechnology, Tung Hai University, Taichung 40722, Taiwan; 11 Department of Palliative Care Unit, Taichung Veterans General Hospital, Taichung 40705, Taiwan; 12 Ph.D. Program in Translational Medicine, Institute of Biomedical Sciences, National Chung Hsing University, Taichung 40220, Taiwan; 13 Rong Hsing Research Center for Translational Medicine, National Chung Hsing University, Taichung 40220, Taiwan

**Keywords:** advanced endometrial cancer, lenvatinib, pembrolizumab, doxorubicin, adverse events

## Abstract

**Background**: The treatment-free interval is a significant predictor of worse prognosis and poor response rates of the second-line treatment in patients with carboplatin and paclitaxel (PT)-pretreated, advanced, or recurrent endometrial cancer (EC). Whether lenvatinib plus pembrolizumab still confers a survival benefit compared with doxorubicin in patients with platinum-free intervals of <6 months remains unclear. **Methods**: This multi-institutional retrospective analysis was performed using de-identified electronic health records from the TriNetX Research Network. Patients with advanced or recurrent ECs who received lenvatinib plus pembrolizumab or doxorubicin within six months of first-line PT were identified. A 1:1 propensity score matching (PSM) was conducted to control for potential confounding variables. Overall survival (OS) and adverse event profile were the primary and secondary outcomes. **Results**: Between January 2018 and February 2024, 130 patients with PT-treated, advanced, or recurrent ECs who received lenvatinib plus pembrolizumab and 122 patients who received doxorubicin at a platinum-free interval of <6 months were identified across 31 healthcare organizations. In the balanced cohort following PSM with 117 patients in each group, treatment with lenvatinib plus pembrolizumab was associated with improved OS compared with treatment with doxorubicin (12.8 vs. 8.2 months, *p* = 0.012, hazard ratio: 0.65, 95% confidence interval: 0.46–0.91). Regarding adverse event analysis, a higher incidence of hypothyroidism and proteinuria was observed with lenvatinib plus pembrolizumab, and more hematological toxicities were observed with doxorubicin. **Conclusions**: in patients with treatment-free intervals of <6 months, lenvatinib plus pembrolizumab still confers improved survival compared with doxorubicin in PT-treated, advanced, or recurrent ECs.

## 1. Introduction

Endometrial cancer (EC) is the most common malignancy of the female reproductive system with an increasing incidence [1]. In 2024, it is estimated that there will be 67,880 new cases of EC diagnosed, and 13,250 patients are expected to die from the disease in the United States [1]. Although the majority of ECs are diagnosed early and have a favorable outcome, the prognosis for advanced or recurrent ECs is generally poor, with a median survival of 12–15 months among patients with measurable disease [2].

Tri-weekly carboplatin plus paclitaxel (PT) has been standardized as a first-line therapy for advanced ECs since 2012. However, the options for second-line systemic treatments have been limited, and no standard of care has been established in managing patients who progressed on platinum-based chemotherapy [3,4]. Regarding the treatment of recurrent ECs, clinicians often consider platinum rechallenge, provided that a reasonably long progression-free interval has passed after first-line PT [5,6]. Nevertheless, the concept of “platinum-free interval” still lacks validation from prospective studies. Doxorubicin, historically used as first-line chemotherapy for advanced ECs, was reported to have a 19–25% response rate for chemo-naïve patients [7]. In patients experiencing short progression-free intervals after first-line PT, platinum rechallenge becomes unfeasible, leaving doxorubicin monotherapy as one of the few viable options.

Pembrolizumab, an anti-programmed death 1 immune checkpoint inhibitor, has demonstrated improved objective response rates (ORRs) of 57% among patients with high micro-satellite instability (MSI-H) or deficient mismatch repair (dMMR) ECs [8]. However, when used as a monotherapy against micro-satellite stable (MSS) ECs, the ORR dropped to 13% [9]. Nevertheless, the KEYNOTE-146/Study-111 reported improved ORRs of 36% with the addition of lenvatinib, an oral multi-kinase inhibitor targeting VEGF receptors 1–3, fibroblast growth factors (FGF) receptors 1–4, platelet-derived growth factors α receptor, RET, and KIT [10,11]. Subsequently, the KEYNOTE-775 study demonstrated that the combination of lenvatinib plus pembrolizumab resulted in improved overall survival (OS) [18.3 vs. 11.4 months, 95% confidence interval (CI) 0.51–0.75] when compared with single-agent doxorubicin or weekly paclitaxel in a cohort of patients with platinum-treated EC without biomarker preselection [12]. As a consequence, lenvatinib plus pembrolizumab was listed as a category 1 recommendation by the National Comprehensive Cancer Network (NCCN) guideline in platinum-pretreated patients with proficient mismatch repair (pMMR), recurrent ECs [13].

Treatment-free interval has been recognized as an important predictor of survival after recurrences [14,15,16]. Response rates to second-line treatments may vary depending on treatment-free intervals, and clinicians may alter their choice of systemic treatment accordingly [17,18,19]. Although the KEYNOTE-775 study has shown improved survival outcomes after lenvatinib plus pembrolizumab, there has been no research evaluating the outcomes of lenvatinib plus pembrolizumab in patients with short treatment-free intervals to date. Whether lenvatinib plus pembrolizumab confers a survival benefit compared with doxorubicin among patients whose disease progressed within six months of first-line PT remains uncertain. Herein, we performed a retrospective analysis based on real-world data of patients with advanced or recurrent ECs who received either pembrolizumab plus lenvatinib or doxorubicin within a treatment-free interval of six months from first-line PT.

## 2. Materials and Methods

This retrospective, multi-institutional study used de-identified data from the TriNetX Research Network (Cambridge, MA, USA). The TriNetX Research Network provides access to the electronic medical records of approximately 111 million patients across 86 healthcare organizations (HCOs) predominantly located in the United States and Europe. The TriNetX platform adheres to the Health Insurance Portability and Accountability Act and has obtained a waiver from the Western Institutional Review Board. All data on the TriNetX platform are displayed in aggregate form, and information is de-identified, thereby ensuring compliance with privacy regulations [20,21].

The data used in this study were acquired from the TriNetX Research Network in March 2024. Study participants were identified using International Classification of Disease (ICD) codes from January 2018 to February 2024. Only individuals aged 18–90 years were included in this study. Advanced EC was characterized by the presence of EC along with ICD codes indicating metastasis to the lymphatic system and distant metastasis. Chemotherapy, immunotherapy, and targeted therapy regimens were identified using RxNorm and Healthcare Common Procedure Coding System (HCPCS) codes. Surgical procedures and history of irradiation were identified using Current Procedural Terminology (CPT) codes. All the codes used in this study are listed in Supplementary Table S1.

Patients must have received at least 3–6 cycles of PT as first-line chemotherapy before initiating second-line treatments. The interval between first- and second-line treatments was restricted to a maximum of six months. The TriNetX-curated term “chemotherapy line 2” was assigned to both designated treatment groups to avoid misclassifications. The index event in the lenvatinib plus pembrolizumab group was defined as the start date of lenvatinib plus pembrolizumab and likewise for doxorubicin. Patients who received pembrolizumab or lenvatinib before and after the initiation of doxorubicin were excluded. Histological findings of carcinosarcoma and sarcoma were excluded from the analysis. Additionally, patients who underwent a major operation within 3 weeks before starting lenvatinib plus pembrolizumab or doxorubicin were excluded.

The main outcome measure was the median OS for both lenvatinib plus pembrolizumab and doxorubicin groups. The primary endpoint was estimated from the date of the index event in each treatment group to the date of death. Patients were censored based on their final observations in their records. Our secondary outcome was the adverse events in both groups. Hematological, hepatic, and thyroid profiles and systemic adverse events were assessed from the date of the index event. Treatment toxicities were graded according to the Common Terminology Criteria for Adverse Events version 5.0 (CTCAE v5.0) [22].

All statistical analyses were performed using the TriNetX platform in real-time. The statistics of the TriNetX platform were performed by running a suite of tests using R’s Survival package v3.2-3 and comparing the numbers with output from SAS version 9.4 to validate the results. The baseline characteristics between the treatment groups were compared using the Pearson chi-squared test. A 1:1 propensity score matching (PSM) was performed in order to reduce potential confounders between the treatment groups. Matching included covariates such as age, history of irradiation, and the presence of regional or distant metastasis. We utilized a greedy nearest-neighbor matching algorithm with a 0.1 pooled standard deviation caliper. Hence, patients with disparate propensity scores were not paired. In the newly generated cohort following PSM, survival outcomes were estimated using the log-rank test to compare OS between the treatment groups, and hazard ratios (HRs) were generated using Cox regression. All statistical significance in this study was established with a two-sided *p*-value < 0.05. 

## 3. Results

A total of 17,014 patients aged 18–90 years with advanced or recurrent ECs were identified across 31 HCOs between January 2018 and February 2024. After applying the inclusion and exclusion criteria, 456 patients who received 3–6 cycles of first-line PT prior to their designated treatment were identified. After restricting the treatment-free interval to a maximum of six months, we identified 130 patients who received lenvatinib plus pembrolizumab and 122 patients who received doxorubicin as second-line treatments. The median follow-up time was 10.1 and 8.1 months, respectively. The baseline characteristics of both groups before and after PSM are summarized in Table 1. In the unmatched cohort, a higher percentage of patients were white, and a higher prevalence of chronic heart disease was observed among patients who received doxorubicin. 

**Figure 1:**
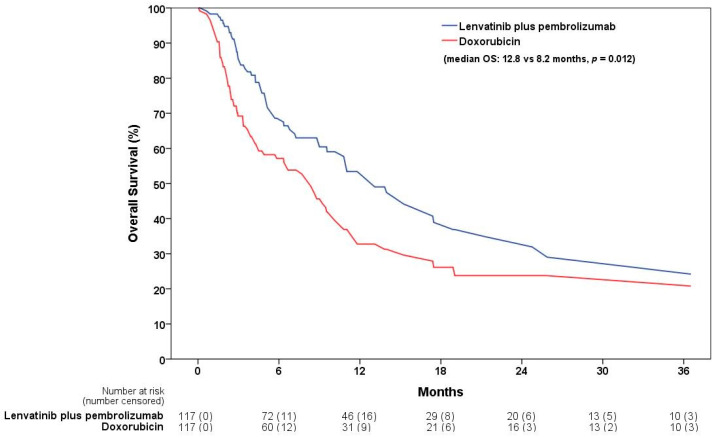
The 3-year OS between the lenvatinib plus pembrolizumab and doxorubicin groups after propensity score matching. OS, overall survival.

Following 1:1 PSM, the matched cohort contained 117 patients each in the lenvatinib plus pembrolizumab and doxorubicin groups. The matched cohort was well balanced, and the distribution of propensity scores before and after matching was displayed using a kernel density estimation plot (Supplementary Figure S1). A total of 58 patients died in the lenvatinib plus pembrolizumab group and 72 in the doxorubicin group, estimated three years after the index event. Lenvatinib plus pembrolizumab was associated with an improved OS when compared with doxorubicin (median OS: 12.8 vs. 8.2 months, *p* = 0.012; HR: 0.65, 95% CI: 0.46–0.91) (Table 2) (Figure 1). Regarding adverse event analysis, hematological toxicities were more common in the doxorubicin group. A higher risk of developing neutropenia (9% vs. 23%, *p* = 0.005), grade 3 anemia (39% vs. 56%, *p* = 0.009), grade 3 neutropenia (9% vs. 21%, *p* = 0.017), and grade 3 thrombocytopenia (9% vs. 18%, *p* = 0.034) was observed. In contrast, proteinuria and hypothyroidism were more prevalent in patients who received lenvatinib plus pembrolizumab, with incidence rates of 31% vs. 16% and 37% vs. 18%, respectively. The risks of liver enzyme abnormalities, hypertension, diarrhea, fatigue, arthralgia, nausea, and vomiting were comparable between the two groups. A comparison of adverse events is shown in Table 3.

## 4. Discussion

In this retrospective, multi-institutional study using real-world data acquired from the TriNetX platform, we found that the combination of lenvatinib plus pembrolizumab was associated with improved OS when compared with doxorubicin monotherapy in patients with advanced or recurrent ECs and treatment-free intervals of <6 months from first-line PT (OS: 12.8 vs. 8.2 months; HR: 0.65; 95% CI: 0.46–0.91). Adverse event analysis showed a higher incidence and severity of hematologic toxicities among patients who received doxorubicin, whereas patients who received lenvatinib plus pembrolizumab exhibited increased instances of proteinuria and hypothyroidism. These findings are consistent with those reported in previous studies [11,12,23,24,25,26].

The treatment-free interval after first-line PT has been shown to be strongly correlated with survival. A pooled analysis from five prospective randomized trials conducted by the Gynecologic Oncology Group (GOG) with 586 patients who had advanced or recurrent EC after first-line chemotherapy revealed that the interval between the first primary chemotherapy and recurrence was the most significant prognostic factor for second-line chemotherapy [14]. The risk of death was significantly reduced by 30% in patients with progression-free intervals over six months compared with those with intervals under six months. Additionally, the median OS for second-line treatment also increased by five months (10 vs. 5 months). The response rates were invariably poor among patients with treatment-free intervals under three months, regardless of platinum or non-platinum agents received as second-line chemotherapy (ORR < 10% in both groups) [14]. A retrospective study with platinum-based first-line treatment revealed that platinum rechallenge was ineffective in patients with treatment-free intervals of <6 months [17]. Another report from a single-centered study showed an 11.4% response rate and OS of 10 months among patients who underwent platinum rechallenge within a platinum-free interval of <6 months [27]. As a result, before the emergence of immuno-oncology, for patients with short platinum-free intervals where platinum rechallenge is rendered no longer desirable, clinicians were left with limited chemotherapeutic options.

The majority of studies on single-agent chemotherapies as second-line treatments were conducted over a decade ago and consistently presented poor efficacies, with reported ORRs of approximately 5–15% [10,28,29,30,31,32,33,34,35,36,37,38,39,40,41,42] (Table 4). In practice, a preference for selecting doxorubicin over paclitaxel and other regimens for patients with platinum-pretreated ECs has been observed [6,12]. This preference was also evident in the composition of the chemotherapy arm, which was determined by physicians’ preference in the KEYNOTE-775 study [12]. Out of the 388 patients in the chemotherapy arm, 289 received doxorubicin, while only 99 received paclitaxel. Doxorubicin was reported to have a 19–25% response rate when used in chemotherapy-naïve patients [7,28]. However, doxorubicin monotherapy has been reported to have limited efficacies in second-line settings, with ORRs of approximately 10–15% and median OS of 5.8–12.3 months [28,29,30,31,32]. One retrospective study investigating 17 patients with a median treatment-free interval from first-line PT of eight months found that none of the patients responded to second-line doxorubicin monotherapy [30]. On the other hand, a 27% ORR has been historically reported for paclitaxel in paclitaxel-naïve patients [33]. However, considering the short treatment-free interval following first-line PT, whether a 27% ORR can be achieved remains uncertain. These findings highlight the constrained options that clinicians often face when managing patients experiencing disease progression shortly after first-line PT, underscoring the need for a better option.

Pembrolizumab has yielded a 64% ORR and sustained progression-free survival (PFS) among patients with MSI-H or dMMR ECs [11]. Nevertheless, MSI-H or dMMR tumors represent only 16–31% of all ECs [43,44,45]. Subsequently, the KEYNOTE-775 study demonstrated a 31.9% ORR and an OS of 18.3 months among a cohort where 84% of patients had pMMR tumors and received lenvatinib plus pembrolizumab [12]. These results have led to a significant shift in clinical practice regarding the choice of second-line treatments [46]. However, reports on survival outcomes with real-world data have been scarce to date. Two single-center studies in Japan yielded ORRs ranging from 40 to 60%, and PFS between 8.6 and 11.6 months [47,48]. A multi-center study involving 48 pretreated patients, with a median treatment-free interval of 4.3 months, who received lenvatinib plus pembrolizumab, reported a 23.8% ORR [25]. Although not reaching statistical significance, a trend toward a worse PFS was observed in patients with treatment-free intervals of <5 months [25]. The largest cohort to date, comprised of 70 patients treated at the MD Anderson Cancer Center, showed a 36.1% ORR and a median OS of 8.6 months [26]. In our study, among 117 patients receiving lenvatinib plus pembrolizumab with a platinum-free interval of <6 months in the matched cohort, the median OS was significantly improved compared with doxorubicin (12.8 vs. 8.2 months, *p* = 0.012; HR: 0.65, 95% CI: 0.46–0.91). Our survival outcome appeared worse when compared with the data presented in the KEYNOTE-775 study (18.3 vs. 11.4 months, *p* < 0.001; HR: 0.62, 95% CI: 0.51–0.75), in which the treatment-free intervals were not specified [12]. This was consistent with the observations that a shorter treatment-free interval from first-line PT was correlated with a poorer prognosis [14,15,16]. Nonetheless, even in such circumstances, lenvatinib plus pembrolizumab still provided superior outcomes compared with doxorubicin.

Studies have shown that the most common adverse events for lenvatinib plus pembrolizumab were hypertension (59–65%), fatigue (34–65%), diarrhea (43–64%), hypothyroidism (47–59%), proteinuria (23–31%), and arthralgia (23–32%) [12,23,24,25,26]. Although these adverse events were generally considered manageable, up to 70% of patients experienced dose reductions and treatment interruptions, with 33% eventually having to discontinue their treatment due to intolerable toxicities [12]. Despite the high rates of dose reduction in lenvatinib, the treatment response rates appeared unaffected when initiated at a reduced dose (most commonly 14 mg daily) compared with the recommended dose (20 mg daily), with ORRs of 38.3% and 28.6%, respectively [26]. In terms of the toxicity profile, our findings were consistent with that of previous reports, showing a higher incidence of hematologic adverse events in the doxorubicin group, as anticipated.

This study had several limitations. First, our study was limited by the absence of details on histological compositions, dosage, and MMR status between groups. Additionally, all patient diagnoses, laboratory results, adverse events, and treatment histories were identified using the ICD, RxNorm, HCPCS, and CPT coding systems. Therefore, our outcomes may have been affected by misclassification or delays in reporting the mortality data. Furthermore, due to platform limitations, data regarding multivariate cox proportional hazard model cannot be obtained. Nevertheless, our results provide valuable real-world data by comparing the two most frequently used strategies for the management of advanced or recurrent ECs at short treatment-free intervals. The considerable number of patients and a well-matched PSM should strengthen our results.

## 5. Conclusions

Lenvatinib plus pembrolizumab, compared with doxorubicin, was associated with improved OS in patients with advanced or recurrent ECs who had short treatment-free intervals from first-line PT. 

## Figures and Tables

**Table 1:** Baseline characteristics of the lenvatinib plus pembrolizumab and doxorubicin cohorts, before and after PSM.

	Before Match			After Matched		
	Lenvatinib + Pembrolizumab N = 130	Doxorubicin N = 122	*p*-Value	Lenvatinib + Pembrolizumab N = 117	Doxorubicin N = 117	*p*-Value
Age at index *	65.6	65.3		65.9	65.5	
<65 years	56 (43)	52 (43)	0.94	47 (40)	47 (40)	1
≥65 years	74 (57)	70 (57)	0.94	70 (60)	70 (60)	1
Race						
White	69 (53)	84 (69)	0.01	63 (54)	80 (68)	0.02
African American	29 (22)	25 (21)	0.73	25 (21)	25 (21)	1
Asian	10 (8)	10 (8)	0.88	10 (9)	10 (9)	1
Unknown	18 (14)	10 (8)	0.15	10 (9)	10 (9)	1
Diabetes mellitus	42 (32)	32 (26)	0.29	37 (32)	32 (27)	0.47
Cerebral vascular disease	10 (8)	16 (13)	0.16	10 (9)	15 (13)	0.29
Ischemic heart disease	20 (15)	19 (16)	0.97	17 (15)	19 (16)	0.72
History of irradiation *	31 (24)	28 (23)	0.87	28 (24)	27 (23)	0.88
Therapies received before designated treatment						
Carboplatin	130 (100)	122 (100)	NA	117 (100)	117 (100)	NA
Paclitaxel	130 (100)	122 (100)	NA	117 (100)	117 (100)	NA
Bevacizumab	10 (8)	22 (18)	0.01	10 (9)	21 (18)	0.03
Sites of metastasis						
Lymph node	46 (35)	31 (25)	0.09	39 (33)	28 (24)	0.11
Para-aortic LN	18 (14)	18 (15)	0.84	14 (12)	17 (15)	0.56
Pelvic LN	25 (19)	17 (14)	0.26	23 (20)	16 (14)	0.22
Lung	28 (22)	19 (16)	0.22	18 (15)	18 (15)	1
Liver	17 (13)	15 (12)	0.85	14 (12)	14 (12)	1
Peritoneum	59 (45)	57 (47)	0.83	53 (45)	55 (47)	0.79
Colorectal	14 (11)	13 (11)	0.98	14 (12)	13 (11)	0.84

Data are No. (%). Demographic data were obtained from the TriNetX Research Network. The baseline characteristics were documented one day before the index date. Variables marked with * were selected for PSM. Patient counts less than 10 will be rounded up to 10 for display to ensure patient anonymity. PT: carboplatin plus paclitaxel; PSM: propensity score matching; LN: lymph nodes; NA: not available.

**Table 2:** Summary of survival outcomes of lenvatinib plus pembrolizumab compared to doxorubicin.

	Pre-Match Case No.	Case No. after Match	Death	Median Follow-Up (mo)	Median OS (mo)
Lenvatinib plus pembrolizumab	130	117	58	10.1	12.8
Doxorubicin	122	117	72	8.1	8.2

OS: overall survival.

**Table 3:** Adverse events among patients receiving lenvatinib plus pembrolizumab and doxorubicin after propensity score matching.

	Lenvatinib + Pembrolizumab N = 117	Doxorubicin N = 117	*p*-Value
Anemia	104 (89)	108 (92)	0.370
Grade 3 anemia	45 (39)	65 (56)	0.009
Neutropenia	11 (9)	27 (23)	0.005
Grade 3 neutropenia	11 (9)	24 (21)	0.017
Thrombocytopenia	14 (12)	18 (15)	0.447
Grade 3 thrombocytopenia	10 (9)	21 (18)	0.034
Liver toxicity	164 (38)	154 (36)	0.481
Hypothyroidism	43 (37)	21 (18)	0.001
Hypertension	72 (62)	72 (62)	1
Grade 3 hypertension	48 (41)	44 (38)	0.592
Proteinuria	36 (31)	19 (16)	0.009
Diarrhea	30 (26)	18 (15)	0.052
Fatigue	34 (29)	33 (28)	0.885
Nausea and vomiting	59 (50)	56 (48)	0.695
Arthralgia	25 (21)	14 (12)	0.054

**Table 4:** Updates of single-agent treatments for previously treated, advanced, or recurrent endometrial cancer.

Treatment	Case Number	ORR (%)	PFS (mo)	OS (mo)
Lenvatinib [10]	133	14.3%	5.6	10.6
Bevacizumab [42]	52	13.5%	4.2	10.5
Doxorubicin [28,29,30]	132	22% *	NA	NA
	33	12.1%	4.4	8.1
	17	0	2.1	5.8
Liposomal doxorubicin [31,32]	32	9.5%	NA	8.2
	52	11.5% *	NA	10.9
Ixabepilone [38]	223	15.2%	3.4	10.9
Paclitaxel [33,34]	44	27.3% *	NA	10.3
	15	26.7% ^‡^	NA	NA
Docetaxel [35]	26	7.7%	2.0	6.4
Oxaliplatin [36]	52	13.5%	NA	NA
Topotecan [37]	22	9%	NA	NA
Gemcitabine [39]	23	4%	1.7	NA
Ifosfamide [40,41]	16	0%	NA	NA
	91	25% ^†^	3.6	8.4

* The study was performed on paclitaxel-naïve patients. † The study was performed as on patients with carcinosarcoma as first-line treatment. ‡ Heterogenous compositions of prior cytotoxic therapies were used, including those who are paclitaxel-naïve. ORR: objective response rate; PFS: progression-free survival; OS: overall survival; NA: not available.

## Data Availability

The data were acquired using the TriNetX platform in this study. Researchers can identify similar cohorts by applying the inclusion and exclusion criteria described in the Methods section. However, TriNetX is a live network that frequently updates with new data; subsequent queries may exhibit mild variations. Data may be obtained from a third party (TriNetX); however, a data-sharing agreement may be required, and data access fees may be incurred.

## Supplementary Materials

The following supporting information can be downloaded at: https://www.mdpi.com/article/10.3390/jcm13195670/s1, Table S1: Details of diagnostic and procedural query codes used in our analysis; Figure S1: Distribution of propensity score matching before and after matching for the lenvatinib plus pembrolizumab and doxorubicin groups.

## References

[1] Siegel R.L., Giaquinto A.N., Jemal A. (2024). Cancer statistics, 2024. CA Cancer J. Clin..

[2] Brooks R.A., Fleming G.F., Lastra R.R., Lee N.K., Moroney J.W., Son C.H., Tatebe K., Veneris J.L. (2019). Current recommendations and recent progress in endometrial cancer. CA Cancer J. Clin..

[3] Miller D.S., Filiaci V.L., Mannel R.S., Cohn D.E., Matsumoto T., Tewari K.S., DiSilvestro P., Pearl M.L., Argenta P.A., Powell M.A. (2020). Carboplatin and Paclitaxel for Advanced Endometrial Cancer: Final Overall Survival and Adverse Event Analysis of a Phase III Trial (NRG Oncology/GOG0209). J. Clin. Oncol..

[4] Fleming G.F. (2015). Second-Line Therapy for Endometrial Cancer: The Need for Better Options. J. Clin. Oncol..

[5] Nagao S., Nishio S., Michimae H., Tanabe H., Okada S., Otsuki T., Tanioka M., Fujiwara K., Suzuki M., Kigawa J. (2013). Applicability of the concept of “platinum sensitivity” to recurrent endometrial cancer: The SGSG-012/GOTIC-004/Intergroup study. Gynecol. Oncol..

[6] Heffernan K., Nikitas F.S., Shukla U., Camejo H.S., Knott C. (2022). Previously treated recurrent or advanced endometrial cancer in England: A real-world observational analysis. Gynecol. Oncol..

[7] Thigpen J.T., Brady M.F., Homesley H.D., Malfetano J., DuBeshter B., Burger R.A., Liao S. (2004). Phase III trial of doxorubicin with or without cisplatin in advanced endometrial carcinoma: A gynecologic oncology group study. J. Clin. Oncol..

[8] Marabelle A., Le D.T., Ascierto P.A., Di Giacomo A.M., De Jesus-Acosta A., Delord J.P., Geva R., Gottfried M., Penel N., Hansen A.R. (2020). Efficacy of Pembrolizumab in Patients with Noncolorectal High Microsatellite Instability/Mismatch Repair-Deficient Cancer: Results from the Phase II KEYNOTE-158 Study. J. Clin. Oncol..

[9] Ott P.A., Bang Y.J., Berton-Rigaud D., Elez E., Pishvaian M.J., Rugo H.S., Puzanov I., Mehnert J.M., Aung K.L., Lopez J. (2017). Safety and Antitumor Activity of Pembrolizumab in Advanced Programmed Death Ligand 1-Positive Endometrial Cancer: Results From the KEYNOTE-028 Study. J. Clin. Oncol..

[10] Vergote I., Powell M.A., Teneriello M.G., Miller D.S., Garcia A.A., Mikheeva O.N., Bidzinski M., Cebotaru C.L., Dutcus C.E., Ren M. (2020). Second-line lenvatinib in patients with recurrent endometrial cancer. Gynecol. Oncol..

[11] Makker V., Taylor M.H., Aghajanian C., Oaknin A., Mier J., Cohn A.L., Romeo M., Bratos R., Brose M.S., DiSimone C. (2020). Lenvatinib Plus Pembrolizumab in Patients with Advanced Endometrial Cancer. J. Clin. Oncol..

[12] Makker V., Colombo N., Casado Herráez A., Santin A.D., Colomba E., Miller D.S., Fujiwara K., Pignata S., Baron-Hay S., Ray-Coquard I. (2022). Study 309–KEYNOTE-775 Investigators. Lenvatinib plus Pembrolizumab for Advanced Endometrial Cancer. N. Engl. J. Med..

[13] National Comprehensive Cancer Network Uterine Neoplasms. Version 2. 2024. https://www.nccn.org/professionals/physician_gls/pdf/uterine.pdf.

[14] Moore K.N., Tian C., McMeekin D.S., Thigpen J.T., Randall M.E., Gallion H.H. (2010). Does the progression-free interval after primary chemotherapy predict survival after salvage chemotherapy in advanced and recurrent endometrial cancer? A Gynecologic Oncology Group ancillary data analysis. Cancer.

[15] Ueda Y., Matsumura Y., Egawa-Takata T., Miyake T., Miyatake T., Yoshino K., Fujita M., Matsuzaki S., Yokoyama T., Miyoshi Y. (2010). Disease-free interval after primary treatment predicts prognosis of recurrent endometrial carcinoma. Anticancer. Res..

[16] Garside J., Shen Q., Westermayer B., van de Ven M., Kroep S., Chirikov V., Juhasz-Böss I. (2023). Association Between Intermediate End Points, Progression-free Survival, and Overall Survival in First-line Advanced or Recurrent Endometrial Cancer. Clin. Ther..

[17] Ueda Y., Miyake T., Egawa-Takata T., Miyatake T., Matsuzaki S., Yokoyama T., Yoshino K., Fujita M., Enomoto T., Kimura T. (2011). Second-line chemotherapy for advanced or recurrent endometrial carcinoma previously treated with paclitaxel and carboplatin, with or without epirubicin. Cancer Chemother. Pharmacol..

[18] Shimamoto K., Saito T., Okadome M., Shimokawa M. (2014). Prognostic significance of the treatment-free interval in patients with recurrent endometrial cancer. Eur. J. Obstet. Gynecol. Reprod. Biol..

[19] Matoda M., Omatsu K., Yamamoto A., Nomura H., Tanigawa T., Kawamata Y., Kato K., Umayahara K., Takeshima N. (2014). Importance of platinum-free interval in second-line chemotherapy for advanced or recurrent endometrial cancer. Eur. J. Gynaecol. Oncol..

[20] The TriNetX Publication Guidelines. 5 June 2023. https://trinetx.com/real-world-resources/publications/trinetx-publication-guidelines/.

[21] Evans L., London J.W., Palchuk M.B. (2021). Assessing real-world medication data completeness. J. Biomed. Inf..

[22] US Department of Health and Human Services, National Institutes of Health, National Cancer Institute Common Terminology Criteria for Adverse Events (CTCAE) Version 5. https://ctep.cancer.gov/protocoldevelopment/electronic_applications/docs/CTCAE_v5_Quick_Reference_5x7.pdf.

[23] Makker V., Taylor M.H., Oaknin A., Casado Herraez A., Orlowski R., Dutta L., Ren M., Zale M., O’Malley D.M. (2021). Characterization and Management of Adverse Reactions in Patients with Advanced Endometrial Carcinoma Treated with Lenvatinib Plus Pembrolizumab. Oncologist.

[24] Makker V., Colombo N., Casado Herráez A., Monk B.J., Mackay H., Santin A.D., Miller D.S., Moore R.G., Baron-Hay S., Ray-Coquard I. (2023). Lenvatinib Plus Pembrolizumab in Previously Treated Advanced Endometrial Cancer: Updated Efficacy and Safety from the Randomized Phase III Study 309/KEYNOTE-775. J. Clin. Oncol..

[25] Kim J., Noh J.J., Lee T.K., Kim S.I., Lee J.Y., Lee J.W., Kim J.W. (2022). Real-world experience of pembrolizumab and lenvatinib in recurrent endometrial cancer: A multicenter study in Korea. Gynecol. Oncol..

[26] How J.A., Patel S., Fellman B., Lu K.H., Hwu P., Ramondetta L.M., Westin S.N., Fleming N.D., Soliman P.T., Jazaeri A.A. (2021). Toxicity and efficacy of the combination of pembrolizumab with recommended or reduced starting doses of lenvatinib for treatment of recurrent endometrial cancer. Gynecol. Oncol..

[27] Yasunaga M., Yahata H., Okugawa K., Hori E., Hachisuga K., Maenohara S., Kodama K., Yagi H., Ohgami T., Onoyama I. (2023). Decision-making for Subsequent Therapy for Patients with Recurrent or Advanced Endometrial Cancer Based on the Platinum-free Interval. Am. J. Clin. Oncol..

[28] Thigpen J.T., Blessing J.A., DiSaia P.J., Yordan E., Carson L.F., Evers C. (1994). A randomized comparison of doxorubicin alone versus doxorubicin plus cyclophosphamide in the management of advanced or recurrent endometrial carcinoma: A Gynecologic Oncology Group study. J. Clin. Oncol..

[29] Moreira E., Paulino E., Ingles Garces Á.H., Fontes Dias M.S., Saramago M., de Moraes Lino da Silva F., Thuler L.C.S., de Melo A.C. (2018). Efficacy of doxorubicin after progression on carboplatin and paclitaxel in advanced or recurrent endometrial cancer: A retrospective analysis of patients treated at the Brazilian National Cancer Institute (INCA). Med. Oncol..

[30] Makker V., Hensley M.L., Zhou Q., Iasonos A., Aghajanian C.A. (2013). Treatment of advanced or recurrent endometrial carcinoma with doxorubicin in patients progressing after paclitaxel/carboplatin: Memorial Sloan-Kettering Cancer Center experience from 1995 to 2009. Int. J. Gynecol. Cancer..

[31] Muggia F.M., Blessing J.A., Sorosky J., Reid G.C. (2002). Phase II trial of the pegylated liposomal doxorubicin in previously treated metastatic endometrial cancer: A Gynecologic Oncology Group study. J. Clin. Oncol..

[32] Homesley H.D., Blessing J.A., Sorosky J., Reid G., Look K.Y. (2005). Phase II trial of liposomal doxorubicin at 40 mg/m^2^ every 4 weeks in endometrial carcinoma: A Gynecologic Oncology Group Study. Gynecol. Oncol..

[33] Lincoln S., Blessing J.A., Lee R.B., Rocereto T.F. (2003). Activity of paclitaxel as second-line chemotherapy in endometrial carcinoma: A Gynecologic Oncology Group study. Gynecol. Oncol..

[34] Homesley H.D., Meltzer N.P., Nieves L., Vaccarello L., Lowendowski G.S., Elbendary A.A. (2008). A phase II trial of weekly 1-hour paclitaxel as second-line therapy for endometrial and cervical cancer. Int. J. Clin. Oncol..

[35] Garcia A.A., Blessing J.A., Nolte S., Mannel R.S., Gynecologic Oncology Group (2008). A phase II evaluation of weekly docetaxel in the treatment of recurrent or persistent endometrial carcinoma: A study by the Gynecologic Oncology Group. Gynecol. Oncol..

[36] Fracasso P.M., Blessing J.A., Molpus K.L., Adler L.M., Sorosky J.I., Rose P.G. (2006). Phase II study of oxaliplatin as second-line chemotherapy in endometrial carcinoma: A Gynecologic Oncology Group study. Gynecol. Oncol..

[37] Miller D.S., Blessing J.A., Lentz S.S., Waggoner S.E. (2002). A phase II trial of topotecan in patients with advanced, persistent, or recurrent endometrial carcinoma: A gynecologic oncology group study. Gynecol. Oncol..

[38] McMeekin S., Dizon D., Barter J., Scambia G., Manzyuk L., Lisyanskaya A., Oaknin A., Ringuette S., Mukhopadhyay P., Rosenberg J. (2015). Phase III randomized trial of second-line ixabepilone versus paclitaxel or doxorubicin in women with advanced endometrial cancer. Gynecol. Oncol..

[39] Tait D.L., Blessing J.A., Hoffman J.S., Moore K.N., Spirtos N.M., Lachance J.A., Rotmensch J., Miller D.S. (2011). A phase II study of gemcitabine (gemzar, LY188011) in the treatment of recurrent or persistent endometrial carcinoma: A gynecologic oncology group study. Gynecol. Oncol..

[40] Pawinski A., Tumolo S., Hoesel G., Cervantes A., van Oosterom A.T., Boes G.H., Pecorelli S. (1999). Cyclophosphamide or ifosfamide in patients with advanced and/or recurrent endometrial carcinoma: A randomized phase II study of the EORTC Gynecological Cancer Cooperative Group. Eur. J. Obstet. Gynecol. Reprod. Biol..

[41] Homesley H.D., Filiaci V., Markman M., Bitterman P., Eaton L., Kilgore L.C., Monk B.J., Ueland F.R., Gynecologic Oncology Group (2007). Phase III trial of ifosfamide with or without paclitaxel in advanced uterine carcinosarcoma: A Gynecologic Oncology Group Study. J. Clin. Oncol..

[42] Aghajanian C., Sill M.W., Darcy K.M., Greer B., McMeekin D.S., Rose P.G., Rotmensch J., Barnes M.N., Hanjani P., Leslie K.K. (2011). Phase II trial of bevacizumab in recurrent or persistent endometrial cancer: A Gynecologic Oncology Group study. J. Clin. Oncol..

[43] Ryan N.A.J., Glaire M.A., Blake D., Cabrera-Dandy M., Evans D.G., Crosbie E.J. (2019). The proportion of endometrial cancers associated with Lynch syndrome: A systematic review of the literature and meta-analysis. Genet. Med..

[44] Bonneville R., Krook M.A., Kautto E.A., Miya J., Wing M.R., Chen H.Z., Reeser J.W., Yu L., Roychowdhury S. (2017). Landscape of Microsatellite Instability Across 39 Cancer Types. JCO Precis. Oncol..

[45] Fan C.T., Hsu S.T., Sun L., Hwang S.F., Liu C.K., Shih Y.H., Chen M.J., Li H.N., Wang J.S., Wen M.C. (2023). Improved progression-free survival associated with tumor-infiltrating lymphocytes in high-grade endometrial cancer. J. Clin. Med..

[46] Giannone G., Castaldo D., Tuninetti V., Scotto G., Turinetto M., Valsecchi A.A., Bartoletti M., Mammoliti S., Artioli G., Mangili G. (2022). Management of Metastatic Endometrial Cancer: Physicians’ Choices Beyond the First Line. A MITO Survey. Front. Oncol..

[47] Chiba Y., Kagabu M., Osakabe M., Ito R., Sato S., Takatori E., Kaido Y., Nagasawa T., Shoji T., Yanagawa N. (2024). A single-institution retrospective exploratory analysis on the effectiveness and safety of lenvatinib plus pembrolizumab for advanced endometrial cancer: Insights from ProMisE molecular classification system. Jpn. J. Clin. Oncol..

[48] Tochigi M., Shigeta S., Shimada M., Miyahara S., Hasegawa-Minato J., Shibuya Y., Ishibashi M., Hashimoto C., Tokunaga H., Yaegashi N. (2024). Lenvatinib plus Pembrolizumab Combination Therapy for Advanced or Recurrent Endometrial Cancer: A Single-Center, Retrospective Analysis. Tohoku J. Exp. Med..

