# Supplementary material for: Lenvatinib Plus Pembrolizumab versus Doxorubicin for Advanced or Recurrent Endometrial Cancer with Short Treatment-Free Intervals Following First-Line Carboplatin Plus Paclitaxel
Source: J Clin Med. 2024 Sep 24;13(19):5670. doi: 10.3390/jcm13195670 (PMC11476733; doi:10.3390/jcm13195670)

Supplementary Materials

Supplementary Table S1. Details of diagnostic and procedural query codes used in our analysis. The codes we used to identify patient diagnosis, medications, and lab results from the International Classification of Disease 10th Clinical Modification codes (ICD-10-CM), RxNorm codes, Healthcare Common Procedure Coding System (HCPCS) codes, Current Procedural Terminology (CPT) codes, Systematized Nomenclature of Medicine (SNOMED), and TriNetX curated codes were enlisted in the table below.

Diagnosis		
Endometrial cancer	ICD-10-CM: C54·1	Malignant neoplasm of endometrium
Sites of metastasis	ICD-10-CM: C78	Secondary malignant neoplasm of respiratory and digestive organs
	ICD-10-CM: C79	Secondary malignant neoplasm of other and unspecified sites
	ICD-10-CM: C77·0	Secondary and unspecified malignant neoplasm of lymph nodes of head, face and neck
	ICD-10-CM: C77·1	Secondary and unspecified malignant neoplasm of intra-thoracic lymph nodes
	ICD-10-CM: C77·2	Secondary and unspecified malignant neoplasm of intra-abdominal lymph nodes
	ICD-10-CM: C77·3	Secondary and unspecified malignant neoplasm of axilla and upper limb lymph nodes
	ICD-10-CM: C77·4	Secondary and unspecified malignant neoplasm of inguinal and lower limb lymph nodes
	ICD-10-CM: C77·5	Secondary and unspecified malignant neoplasm of intra-pelvic lymph nodes
	ICD-10-CM: C77·8	Secondary and unspecified malignant neoplasm of lymph nodes of multiple regions
	ICD-10-CM: C77·9	Secondary and unspecified malignant neoplasm of lymph node, unspecified

Medications		
Carboplatin	RxNorm:40048	Carboplatin
	HCPCS:J9045	Carboplatin
Paclitaxel	RxNorm:56946	Paclitaxel
	HCPCS:J9267	Paclitaxel
Docetaxel	RxNorm:72962	Docetaxel
	HCPCS:J9171	Docetaxel
Doxorubicin	RxNorm:3639	Doxorubicin
	HCPCS:J9000	Doxorubicin
Pembrolizumab	RxNorm:1547545	Pembrolizumab
	HCPCS:J9271	Pembrolizumab
Lenvatinib	RxNorm:1603296	Lenvatinib
Bevacizumab	RxNorm:253337	Bevacizumab
Procedures		
First line chemotherapy	TNX:100211	Chemotherapy, Line 1 (VA Class)
Second line chemotherapy	TNX:100212	Chemotherapy, Line 2 (VA Class)
Radiotherapy	TNX:1010843	Radiation oncology treatment
Outcomes (Diagnosis & Lab exams with LOINC)		
Deceased		
Anemia	ICD-10-CM: D64<br>LOINC: 718-7	Anemia: hemoglobin 10.0 to 12.0 g/dL<br>Grade 3 anemia: hemoglobin ≦8.0 g/dL
Neutropenia	ICD-10-CM: D70<br>LOINC:26499-4	Neutropenia: 1,500 to 2,500/mm^3<br>Grade 3 neutropenia: 500 to 1,000/mm^3
Thrombocytopenia	ICD-10-CM: D69.6<br>LOINC:26515-7	Thrombocytopenia: 75,000 to 100,000/mm^3<br>Grade 3 thrombocytopenia: 25,000 to 50,000/mm^3
Liver toxicity	LOINC: 1920-8, 1742-6	AST > 40 U/L<br>ALT > 56 U/L
Hypothyroidism	ICD-10-CM: E03.9	

Hypertension	ICD-10-CM: I10<br>TNX: 9085, 9086	Grade 3 hypertension: Systolic BP≧ 160mmHg or Diastolic ≧100mmHg
Proteinuria	ICD-10-CM: R80	Presence of protein in urine
Diarrhea	ICD-10-CM: R19.7	
Fatigue	ICD-10-CM: R53.83	
Nausea and vomiting	ICD-10-CM: R11	
Arthralgia	ICD-10-CM: M25.5	
Ischemic heart disease	ICD-10: I20-I25	
Cerebrovascular disease	ICD-10: I60-69	
Diabetes mellitus	ICD-10: E08-E13	

Supplementary Figure S1. Distribution of propensity score matching before and after matching for the lenvatinib plus pembrolizumab and doxorubicin groups.

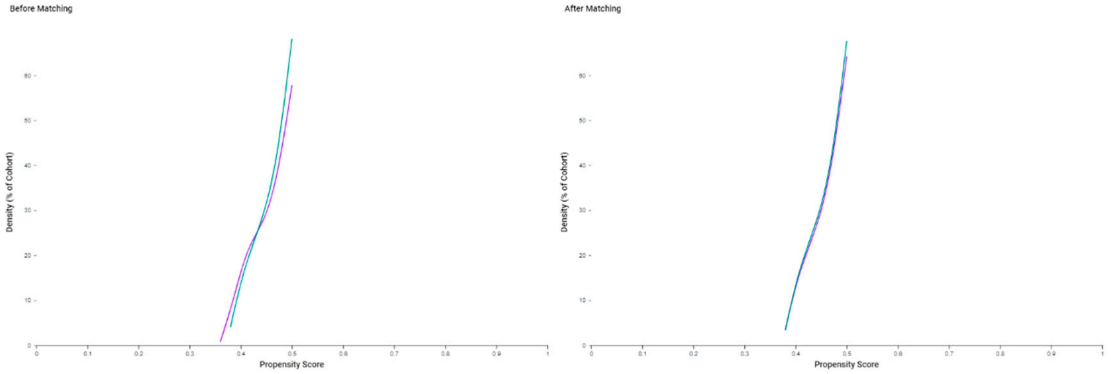

Supplement: Supplementary file 1 [file jcm-13-05670-s001.zip › jcm-3115613-supplementary.pdf]
